# New Zealand’s Drug Development Industry

**DOI:** 10.3390/ijerph10094339

**Published:** 2013-09-13

**Authors:** Michelle Marie Lockhart, Zaheer-Ud-Din Babar, Christopher Carswell, Sanjay Garg

**Affiliations:** 1 School of Pharmacy, Faculty of Medicine and Health Sciences, University of Auckland, Private Bag 92019, Auckland 1010, New Zealand; E-Mail: m.lockhart@auckland.ac.nz; 2 Springer Healthcare, Auckland 0632, New Zealand; E-Mail: Chris.Carswell@springer.com; 3 School of Pharmacy and Medical Sciences, University of South Australia, Adelaide SA 5001, Australia; E-Mail: Sanjay.Garg@unisa.edu.au

**Keywords:** drug development, economic benefits, New Zealand, clinical research, drug discovery

## Abstract

The pharmaceutical industry’s profitability depends on identifying and successfully developing new drug candidates while trying to contain the increasing costs of drug development. It is actively searching for new sources of innovative compounds and for mechanisms to reduce the enormous costs of developing new drug candidates. There is an opportunity for academia to further develop as a source of drug discovery. The rising levels of industry outsourcing also provide prospects for organisations that can reduce the costs of drug development. We explored the potential returns to New Zealand (NZ) from its drug discovery expertise by assuming a drug development candidate is out-licensed without clinical data and has anticipated peak global sales of $350 million. We also estimated the revenue from NZ’s clinical research industry based on a standard per participant payment to study sites and the number of industry-sponsored clinical trials approved each year. Our analyses found that NZ’s clinical research industry has generated increasing foreign revenue and appropriate policy support could ensure that this continues to grow. In addition the probability-based revenue from the out-licensing of a drug development candidate could be important for NZ if provided with appropriate policy and financial support.

## 1. Introduction

The pharmaceutical industry is undergoing considerable change as it seeks to address its declining profitability caused primarily by declining research and development (R&D) productivity and increasing R&D costs. Its challenge is to find alternative sources of innovative compounds and more efficient mechanisms of managing them through the high-risk drug development process. As a result the industry is moving away from its traditional in-house or closed method of drug development [1,2,3,4,5]. The new, more open approach to drug development involves the industry forming alliances and partnerships with smaller companies and academic groups to gain access to innovative compounds and complementary expertise [1,6]. The industry is also outsourcing parts of the R&D process in an attempt to reduce the extraordinary expense of drug development.

The industry’s rapid expansion in their outsourcing of drug discovery and development projects has created significant opportunities and there is increasing competition from countries wanting to capitalise on these [1]. A policy to support its drug development industry is attractive to governments because of the potential benefits of wealth creation, employment, international trade and the desired development of high technology industries [7]. In addition, a viable pharmaceutical industry could reduce a country’s dependency on expensive imported medicines, or provide treatments for their population’s specific medical needs [6,7,8,9].

The barriers to a successful drug development industry include the high R&D costs, knowledge capital required, price competition from emerging economies and unpredictable potential economic benefits [10,11]. The risks of drug development, both technical and financial, are well-known but often underestimated, and the return-on-investment horizon can be more than 20 years [12]. These factors make the development of a local industry a high risk proposition, however this risk may be mitigated by the increasing opportunity created by the pharmaceutical industry outsourcing drug development projects [10].

New Zealand (NZ) has the advantages of a strong biomedical research basis for drug discovery innovations, a resourceful and entrepreneurial society that encourages innovation [13], and a Western culture while being conveniently located in the Asia-Pacific region. The NZ government has invested in science, research and technology as a mechanism to increase the knowledge economy, encourage innovation and support NZ’s best biomedical and drug development research [14,15]. However, many developed and developing countries have also implemented policies to promote local innovation and increase capability in drug development. Therefore, NZ’s challenge is to understand where to position itself in the global drug development industry.

An assessment of existing expertise would allow NZ to define its strengths in drug development and allow it to differentiate itself from competitor countries. There is also a need to understand the enablers and barriers that have influenced the growth of the NZ drug development industry so far and to identify those that could provide support for further industry development. Finally, calculations of the potential economic value that could accrue to NZ from its drug development industry do not appear to have been conducted and an estimation of this value is an important component of an assessment of the viability of this industry. The results of this research into NZ’s drug development industry would assist NZ in maximising the opportunities presented by the current changes in the pharmaceutical industry, as it faces the challenges of finding new sources of innovative compounds and more cost-efficient drug development processes.

The objectives of our research were:

- To develop a theoretical framework for evaluating the drug development industry in NZ
- To critically evaluate the expertise of this industry in NZ
- To identify the enablers and barriers to the use and/or development of NZ expertise and the factors that have allowed this industry to arise
- To assess the potential economic benefits to NZ of policies supporting the drug discovery and development industry

This research was conducted in a manner that involved as much of NZ’s drug development industry as was possible, rather than using an in-depth case study approach of a few selected organisations. The three topic strands of expertise, enablers and barriers, and economic benefits are not mutually exclusive and therefore had the potential to produce complementary findings.

## 2. Methods

Theoretical frameworks were developed to assess the NZ drug development industry and questionnaires were designed to obtain appropriate data from senior representatives of almost all of NZ’s organisations. A separate framework was developed and applied to propose policy models for categorising a country’s strategy, the policies and factors affecting NZ’s industry development, and the policies to support further growth. The potential revenue that could accrue to NZ from the two facets of its drug development industry where it has recognised strengths derived from [1] from the successful development of a NZ-discovered medicine and [2] from the provision of clinical research services to overseas organisations.

### 2.1. Revenue fromthe Successful Development of a NZ-Discovered Medicine

A hypothetical compound was used to calculate the potential revenue to NZ of the successful development of a NZ-discovered medicine. This method was chosen because of the confidentiality issues of using an actual development candidate and we do not have access to confidential pharmaceutical industry information. Our calculations on a hypothetical compound were based on data from previous research and a summary of the assumptions made for our calculation of revenue to NZ are provided in Table 1.

The out-licence of a promising drug discovery candidate could provide income as upfront and royalty payments for an academic medicinal chemistry centre to expand and undertake more commercially directed research alongside their publicly funded research. We used an average cost of a medicinal chemist or biologist of NZD 200,000 ($168,000) to cover salary, rent, equipment and consumables costs [16].

### 2.2. Revenue from Clinical Research

We estimated the revenue to clinical trial sites performing research for the pharmaceutical industry. We used an average per participant payment of NZD 15,000 ($12,600) which was confirmed with several NZ clinical research facilities. It is lower than estimates from the US which may reflect the lower costs of labour and services in NZ. We obtained access to the NZ Ministry of Health databases of applications for clinical trials involving unregistered medicines which provided the number of participants expected at NZ sites and the clinical trial sponsor. We applied an average per participant payment for the 2010/2011 year and reduced it by 3% per year going back to the 1998/1999 year (the earliest year for which the relevant data was available). The revenue to NZ each year from its clinical research activities was estimated by multiplying the number of participants expected from industry-sponsored clinical trial applications each year by the per participant payment for that year. This calculation does not include other trial payments such as set-up fees, ethics application submission, and the costs of the sponsor monitoring and managing the study sites.

**Table 1:** Assumptions for the calculation of potential revenue from drug discovery.

Parameter	Assumption	Basis of the assumption
Timing of out-license deal	Pre-clinical (*i.e*., without clinical data)	N/A
Local ownership when deal agreed	100%	N/A
Upfront payment	$6.5 million	Research by Kessel and Frank [17]
Projected global peak sales	$350 million	N/A
Time of global peak sales	Year 10 after product launch	Data from Danzon and Kim [18], Grabowski [19] and Hoyle [20]
Duration of sales	20 years	Data from Danzon and Kim [18], Grabowski [19] and Hoyle [20]
Sales for Year 1 to Year 20 as a percentage of peak sales	Bell-shaped curve, as described in Table 2	Data from Rasmussen [21] and Cook [22]
Probability that a self-originated compound is approved for sale	16%	Research by DiMasi and Feldman [23]
Average gross profit on sales	50%	Data from Rasmussen [21]
Royalty payments on sales profit	10%	Research by Kessel and Frank [17]

The following exchange rates (for 18 July 2011) were used for our research: NZD 1.00 = $0.84 and AUS 1.00 = $1.07.

## 3. Results and Discussion

### 3.1. Revenue from the Successful Development of a NZ-discovered Medicine

The returns to NZ per year from a compound with peak annual sales of $350 million are provided in Table 2. 

**Table 2:** Revenue from the out-license of a NZ-discovered medicine.

Out-license deal after pre-clinical stage	Percent probability of successful completion	Project sales as percent of peak global sales (%)	Projected sales/milestone payment per year ($ million)	Projected profit (50% of sales)	Projected profit multiplied by percent probability of success	Probability based payments to NZ ($ million)
Upfront Payment	100	N/A	6.500	N/A	6.500	6.500
Successful Phase I	71	N/A	0	N/A	0	0
Successful Phase II	31.95	N/A	0	N/A	0	0
Successful Phase III and registration dossier submitted	20.45	N/A	0	N/A	0	0
Approval of registration dossier	19.02	N/A	0	N/A	0	0
Year 1 sales	16	30	105.000	52.500	8.400	0.840
Year 2 sales	16	40	140.000	70.000	11.200	1.120
Year 3 sales	16	50	175.000	87.500	14.000	1.400
Year 4 sales	16	60	210.000	105.000	16.800	1.680
Year 5 sales	16	70	245.000	122.500	19.600	1.960
Year 6 sales	16	80	280.000	140.000	22.400	2.240
Year 7 sales	16	85	297.500	148.750	23.800	2.380
Year 8 sales	16	90	315.000	157.500	25.200	2.520
Year 9 sales	16	95	332.500	166.250	26.600	2.660
Year 10 sales	16	100	350.000	175.000	28.000	2.800
Year 11 sales	16	90	315.000	157.500	25.200	2.520
Year 12 sales	16	80	280.000	140.000	22.400	2.240
Year 13 sales	16	75	262.500	131.250	21.000	2.100
Year 14 sales	16	70	245.000	122.500	19.600	1.960
Year 15 sales	16	60	210.000	105.000	16.800	1.680
Year 16 sales	16	50	175.000	87.500	14.000	1.400
Year 17 sales	16	40	140.000	70.000	11.200	1.120
Year 18 sales	16	35	122.500	61.250	9.800	0.980
Year 19 sales	16	30	105.000	52.500	8.400	0.840
Year 20 sales	16	25	87.500	43.750	7.000	0.700
Total ($ million)			4,399.000	2,196.250	357.900	41.640

No adjustments (e.g., Net Present Value) have been made because it was assumed that the returns would be invested back into NZ drug discovery almost immediately to fund further research rather than accumulated for future projects. These proceeds to NZ over the average of 30 years from the out-license deal until sales are negligible, would provide total probability-adjusted returns of $41.640 million per discovered medicine. Assuming that only one third was re-invested in building NZ’s drug discovery capability an average of $462,667 per year would fund approximately three additional scientists to research drug discovery projects for 30 years.

Similar calculations using later timings of a licensing-out deal found that the returns to NZ would be 67% higher in total with phase I data and more than two and a half times higher after phase II.

Other analyses were conducted to check the validity and the effects of the following assumptions: peak sales, royalty payment levels, percent probability of approval of the registration dossier, average gross profit on sales and total cumulative sales. A summary of all six analyses is provided in Table 3.

**Table 3:** Summary of sensitivity analyses.

Sensitivity analysis	Detail and total revenue to NZ ($ million)					
	Lower end of the range		Original calculation		Upper end of the range	
	Analysis detail	Revenue to NZ ($ million)	Analysis detail	Revenue to NZ ($ million)	Analysis detail	Revenue to NZ ($ million)
Later out licence deal	N/A	N/A	Pre-clinical	41.640	Post ph I Post ph II	68.347 140.599
Value of peak sales	$50 million	11.520	$350 million	41.640	$1,000 million	106.900
Level of royalty payments	8% of sales profit	33.572	10% of sales profit	41.640	12% of sales profit	47.608
Percent probability of approval of registration dossier	10.0%	28.463	16.0%	41.640	30.0%	72.388
Sales profitability	40% of sales value	33.312	50% of sales value	41.640	60% of sales value	49.968
Cummulative sales	$3,294.4 million	32.894	$4,399.0 million	41.640	$5,278.8 million	48.730

### 3.2. Revenue from Clinical Research

**Figure 1:**
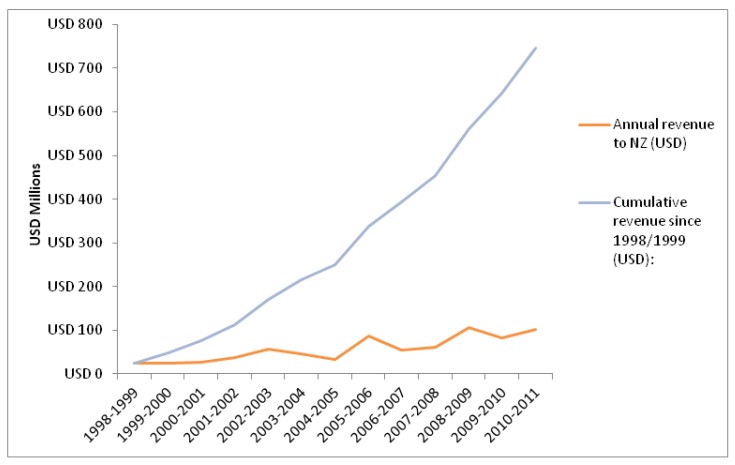
Annual and cumulative revenue from clinical research.

The revenue generated from pharmaceutical industry sponsored clinical trials, based on the expected number of participants in pharmaceutical industry sponsored trials and an average per participant payment to study sites provided $100 M in foreign earnings in 2010/2011. The cumulative revenue since 1998/1999 is estimated at $745 M (see Figure 1).

A sensitivity analysis used a lower per participant payment of $8,400 and an upper level of $21,000 (*i.e*., similar to that published for US sites). The lower payment produced a return to NZ in 2010/2011 of $68 million and cumulative revenues of $497 million since 1998/1999. The upper end of the payment range generated revenue of $170 million in 2010/2011 and cumulative revenues of $1,242 million since 1998/1999.

### 3.3. Discussion

The research results are specific to NZ and cannot be transferred or applied directly to another country. However, the method of assessing the viability of a drug development industry from the three overlapping perspectives of expertise, enablers and barriers, and economic returns could be implemented by another country or region with a similar sized industry to NZ. In addition, the method could be adapted to evaluate the larger drug development industry of a more advanced country. Generally, individual companies in the industry are assessed by commercial organisations and compared with their peers; an assessment of an entire country’s drug development industry has not previously been undertaken, although there has been research into biotechnology industries and clusters.

It is difficult for a country that does not have a strong tradition in pharmaceuticals to create a high technology drug development industry [24]. Despite NZ’s expertise it has been able to grow only a limited industry based on its own discovery compounds. However, it is clear that countries of small economic size but with a high R&D intensity, knowledge intensive labour force and successful local R&D companies can be major contributors to the biotechnology field (e.g., Finland and Sweden). This suggests that smaller countries may benefit from improved co-ordination, and strategies to focus on niche areas may allow more effective knowledge sharing as there are relatively few parties involved [25]. A NZ drug development cluster should be able to benefit from the effective and close connections that are possible due its small size and build on its specific drug discovery and clinical research expertise. At least initially, NZ should concentrate on these niche areas of expertise and not attempt to compete in the areas of drug development where other countries hold an economic or technical advantage; as yet, NZ’s industry is too small.

Our research has found that clinical research generates measurable revenue for NZ and that there is potential revenue to be gained from NZ’s expertise in drug discovery. Our research methodology could be used by other countries with limited resources to estimate their potential revenues from drug discovery and clinical research and to identify the sectors of drug development where it would be the most beneficial for them to focus their efforts. Countries that have limited resources cannot support a fully integrated pharmaceutical industry which is an expensive and risky enterprise. Instead they should initially focus on their niche areas of expertise [24].

Even though the optimum time for an organisation to out-licence a product is after phase II, data from 2008 suggested that approximately 50% of out-licence deals occurred with pre-clinical compounds indicating that many organisations cannot wait until they have sufficient clinical data [26]. Licensing out a drug development candidate is a viable option for an academia-based discovery group that has limited access to funding [27]. Another option to maximise academic expertise is through industry partnerships to fund specific research projects; an example of this model is the collaborative research funding and alliance between GSK and Imperial College London scientists [2].

The probability that a discovery compound is approved for sale is higher if the compound has been licensed-in to the organization and lower for self-originated compounds. Success rates are also affected by the therapeutic class of the compound, with the highest success rates for systemic anti-infectives and lowest for central nervous system products, and antineoplastics having an average success rate. The variability is due to differences in regulatory uncertainties and the level of scientific knowledge [23].

The revenues from an out-licensed product depend primarily on the peak global sales and the timing of the out-license agreement. The assumptions made for our calculations were based on the literature, and our predictions maybe limited by the data publicly available, however even the worst case scenario in Table 3 provides some revenue to re-invest into drug discovery research. The projected sales for a new pharmaceutical are affected by a range of product and market factors, such as the therapeutic indication, market size and competitor compounds, which will be unique to every product. These factors need to be considered on an ongoing basis during the development of a new drug and sales projections revised accordingly [22]. Further calculations on more generally achieved peak sales show that peak sales of $200 million would generate revenues of $26.58 million whereas peak sales of $500 million would provide $56.70 million to New Zealand. Our calculations assumed that the compound was still entirely locally owned when out-licensed and has shown that a compound achieving even modest peak global sales ($350 million) has the potential to produce reasonable returns. The returns could continue for 20 years and provide a drug discovery organisation with stable returns to up-scale its drug discovery capabilities, although the scale of revenue will depend on the success of the compounds.

There is an emerging class of drug discovery organizations which are dependent on successful drug development outcomes and robust intellectual property to flourish [2]. However, even drug discovery organisations with successful projects may struggle to become sustainable in the current economic climate, and profitable ones are usually acquired by a major company. Frequently the contract-only drug discovery model is used as a temporary funding mechanism for the fledgling organisation before expanding into an integrated drug development company [21] with the hope of gaining superior financial returns [24]. This research was to ascertain whether the initial returns would be sufficient to support the first stage of this process *i.e*., the growth of a drug discovery cluster. However, this analysis is not sufficiently comprehensive to take into account other factors such as more detailed costs of staffing and infrastructure, the unpredictability of revenue, and the number of concurrent projects which need to be funded in the expectation of one success.

There are several industry factors that should encourage drug discovery groups that are focused predominantly on small molecule research: (1) the pharmaceutical industry has been downsizing its own drug discovery capability [28]; (2) it needs to rapidly increase its discovery output to maintain its profitability [29]; and (3) the majority of new medicines continue to be small molecules [30,31]. NZ’s research has led to successes primarily with small molecules and many have potential indications in oncology [32]. Oncology is an area of global industry focus as indicated by having the highest number of clinical trials from 2005–2007 [33] and is now the therapeutic area with the highest industry investment [34,35]. It is a challenging indication but the industry’s interest has been encouraged by increased knowledge of cancer mechanisms and relatively favourable reimbursement opportunities. However, oncology is also recognized as a very competitive indication with long development times and high attrition rates which may limit success and return on investment [36].

NZ has been generating significant foreign earnings from its clinical trials industry. Our research calculated that the income accrued from industry sponsored clinical trials of $100 million in 2010/2011 is similar to the upper estimate made of the industry in 2004 [37]. It generally increased over the period studied which is contrary to the popular perception that the NZ industry has been in decline. The value of clinical trials in Australia is estimated to be AUS 450 million per year ($482 million) [38] which is comparable on a *per capita* basis with NZ. While NZ’s size will limit the number of participants and sites it can provide for industry-sponsored clinical trials, it does facilitate rapid review of clinical trial applications through centralised processes. The steady increase in the number of industry-sponsored clinical trials indicates that NZ’s capacity for clinical research is not yet saturated. The increase is predominantly due to the rise in the more challenging phase I studies [39], which is encouraging for a smaller country which is unable to enroll very large numbers of participants into research projects.

## 4. Conclusions

Our analyses have explored the potential value to NZ from two sectors of its drug development industry where it has expertise. NZ’s clinical research industry has generated significant and increasing foreign revenue and appropriate policy support could ensure that this continues to grow. The analyses presented here may simplify a complex situation however NZ’s medicinal chemistry expertise and innovative culture could benefit from further financial and policy support to maximise its potential in drug discovery. Out-licensing drug candidates has the advantage of providing an ongoing revenue stream rather than the fee-for-service revenue generated by clinical research, however increasing NZ’s income from providing clinical research services would likely require less financial outlay. If provided with further support, both sectors of NZ’s drug development industry could provide increased returns and enhance NZ’s expertise in these areas.

This research has contributed to our understanding of three areas of the drug development literature: assessment of a country’s expertise, enablers and barriers to industry development and an estimation of the economic returns. The contributions have been based on NZ’s drug development industry but may be relevant to other countries, particularly those with smaller industries. The research has assessed NZ’s entire drug development industry rather than a detailed case-study involving only a few organisations.

First, the research has identified the expertise of the senior representatives of NZ’s drug development industry as indicated by their length of experience, number of outputs and awards received. There is specific expertise in drug discovery, as indicated by the number of novel compounds that NZ research has identified, and in clinical research, as shown by the increasing number of clinical trials involving unregistered medicines.

Second, from the literature review of policies that countries have used to support their drug development industry, a framework of five different policy categories was developed. This framework was used to propose six policy models to categorise each country’s strategy and to indicate which model NZ has adopted. Further, this provided insights that may assist NZ to learn from other countries that are successfully building a drug development industry. The framework was also used to categorise the policies and factors that NZ’s drug development industry identified as enabling and hindering its development and the policies suggested to further support the industry’s growth. Funding policies, both direct and indirect, have been the most important factors influencing NZ’s industry development and were also the most commonly requested policies to further grow the industry. Specific government funding has supported the growth of expertise and therefore NZ’s reputation for quality medical and clinical research. However, NZ’s total R&D investment, both government and business, is low compared with OECD countries and this issue should be addressed, especially as competitor countries continue to increase their investment. Policies to support the creation of a formal NZ-wide drug development cluster that could share specialised services such a regulatory and legal advice would obviate the need for each NZ drug development company to individually seek or replicate these services. New Zealand’s limited pool of expertise could be augmented by policies to support careers in drug development, promote knowledge sharing and increased alliances with the pharmaceutical industry. The number of NZ-discovered compounds in clinical research has not changed appreciably in the last 8 years and government support is required to increase this number to create a larger portfolio of potential medicines.

Finally, the economic analyses have shown that clinical research provides substantial revenue to NZ and that drug discovery could also provide significant returns. The revenue from pharmaceutical industry-sponsored clinical trials has increased over the last 13 years as NZ expanded its expertise and reputation for high quality research. New Zealand’s clinical trials industry needs to be supported to ensure it remains competitive, despite challenges from an increasing number of countries also offering to conduct industry-sponsored clinical trials. Policies requested by the research participants to improve NZ’s clinical trials environment included more rapid ethical review of applications, streamline the administration required to start a clinical trial and ensure costs remain competitive with overseas. Support in the form of increased funding, career development and facilitation of collaborations, is also required to expand NZ’s drug discovery expertise so that the potential returns can be realised. These returns are dependent primarily on the timing of the out-licence deal and product sales, therefore conducting early phase clinical research before out-licensing the product may increase the revenue to NZ. A proportion of the returns from out-license deals could be reinvested to increase the number of NZ-discovered compounds by employing more research medicinal chemists and biologists. Out-licensing of NZ-discovered compounds has the advantage of potentially providing ongoing revenue to NZ rather than the fee-for-service revenue generated by clinical research, however if provided with further support both sectors of NZ’s industry could provide increased returns.

The results of this research can be utilised in two ways: to increase the global pharmaceutical industry’s awareness of NZ’s expertise and to expand NZ’s own drug development industry. The pharmaceutical industry is meeting the challenge of its declining profitability by changing its approach to drug development and increasingly outsourcing many aspects of the drug development process. The industry is actively seeking new sources of innovation as well as more effective and efficient methods of drug development. New Zealand’s identified expertise, particularly in drug discovery and clinical research, should be co-ordinated by policies to support cluster development, which in turn may enhance local development of NZ-discovered compounds. New Zealand has the ongoing challenge of remaining competitive as it faces increasing competition from countries supporting their innovative drug development industries in an attempt to capitalise on the pharmaceutical industry transformation.
